# Predictors of Acute Mortality After Open Pelvic Fracture: Experience From 37 Patients From A Level I Trauma Center

**DOI:** 10.1007/s00268-020-05675-z

**Published:** 2020-07-06

**Authors:** I-Chuan Tseng, I-Jung Chen, Ying-Chao Chou, Yung-Heng Hsu, Yi-Hsun Yu

**Affiliations:** Department of Orthopedic Surgery, Musculoskeletal Research Center, Chang Gung Memorial Hospital and Chang Gung University, 5, Fu-Hsin St. Kweish, 33302 Tao-Yuan, Taiwan

## Abstract

**Background:**

Open pelvic fractures are caused by high-energy traumas and are accompanied by organ injuries. Despite improvements in pre-hospital care, the acute mortality rate following open pelvic fractures remains high. This study aimed to report experiences in managing open pelvic fractures, identify potential independent predictors that contribute to acute mortality in such patients, and generate a scoring formula to predict mortality rate.

**Methods:**

Open pelvic fracture patients managed during a 42-month period were retrospectively studied. Logistic regression analysis was used to determine predictors of acute mortality. Using the Youden index, threshold values of predictors were selected. Significant predictors were weighted to create a scoring formula. The area under the curve (AUC) was tested in this specific group.

**Results:**

The incidence of open pelvic fractures in all pelvic fractures was 4.9% (37/772), and the overall mortality rate was 21.6% (8/37). All the successfully resuscitated patients entered the reconstruction stage survived and underwent the complete treatment course. Univariate and multivariate logistic regression analyses revealed that the revised trauma score (RTS) was the single independent predictor of acute mortality. A scoring formula was generated following the statistical analysis. The probability of mortality was 0% and 100% when the score was above and below −2, respectively. This model predicted mortality with an AUC of 0.948 (95% confidence interval 0.881–1.000, *P* < 0.01).

**Conclusion:**

The RTS may be a potential predictor of acute mortality in open pelvic fracture patients. Further work would be required to validate the clinical efficacy of the generated scoring formula.

## Introduction

Management of pelvic fractures remains challenging for orthopedic surgeons. Difficulties include not only the complexity of the fracture pattern, but also injuries in associated major organs. A pelvic fracture followed by vital organ injuries, such as blunt chest and abdominal trauma and great vessel damages, could lead to a patient’s death [1, 2]. Previous studies have demonstrated that primary causes of death in patients with pelvic fractures are often injuries, and not fractures themselves [3, 4]. However, it is important to note that such studies have largely focused on injuries in closed pelvic fractures.

Indeed, compared to closed ones, open pelvic fractures are less common [5] but can have much higher mortality rate of almost 50% [6]. While this number has significantly decreased thanks to improvements in pre-hospital care, resuscitation protocols, damage control procedures, and multidisciplinary teamwork [7–9], acute mortality rate in open pelvic fractures still remains high compared to other orthopedic injuries [3, 9]. Previous studies have shown factors such as impact of a delayed diversional colostomy could contribute to this figure [10–12]. However, this delayed fecal diversion is likely to give rise to infection and sepsis, which in turn lead to death in the late stage. There is still little discussion in the literature regarding reasons for mortality during resuscitation.

Therefore, this study aimed to report a series of patients with open pelvic fractures resuscitated and managed at a Level I trauma center, where patients’ details were collected and analyzed. We hypothesized that certain potential predictors that could contribute to acute mortality after open pelvic fractures can be identified. In addition, a score formula was generated as a result of statistical analysis to predict mortality risk among open pelvic fracture patients during triage.

## Materials and methods

We retrospectively collected the data of patients with open pelvic fractures between January 2014 and June 2018 from the registration database of the Level I trauma medical center. Patient data were meticulously reviewed under the approval of the Institutional Reviewing Board of the hospital (IRB No.: 201900569B0).

Patients who arrived with closed pelvic fractures were resuscitated and managed according to our established protocol that was based on the Advanced Trauma Life Support Guidelines. When patients with closed pelvic fractures developed shock, the blood transfusion protocol, designed as 1:1:1 PRBC to FFP to platelet, was followed. Furthermore, when they were unresponsive to fluids and blood resuscitation, transarterial embolization was performed as the priority resuscitation procedure to stop retroperitoneal bleeding.

For patients with open pelvic fractures, the priority changed from resuscitation to debridement, gauze packing of the open surgical wound and in case of pelvic instability, external fixation. The blood transfusion protocol was similar to that in closed pelvic fractures. If necessary during damage control orthopedics in open pelvic fractures, life-saving operations, such as thoracotomy and laparotomy, can also be performed simultaneously or sequentially. Once damage control procedures had been performed, the patient was sent to the intensive care unit for monitoring. The packed gauze was usually removed 24 h after the surgery. Timing for other surgeries depended on the condition of the patient. Pelvic osteosynthesis was performed as soon as possible once the patient’s hemodynamical status had been stabilized. While diversional colostomy is not a routine procedure for these patients, it was necessary for Faringer zone I injury patients [8], usually within 48 h after the trauma.

In addition to patients’ data, several classifications and score systems were used to determine predictors of mortality after open pelvic fracture. Trauma scores such as the Injury Severity Score (ISS), New Injury Severity Score (NISS), and Revised Trauma Score (RTS), which are generally applied to patients after major blunt trauma, were used in this current study as well. An Arbeitsgemeinschaft für Osteosynthesefragen (AO) classification for pelvic fracture was implemented to determine the stability of pelvic ring injury. Three classification systems relevant to open fractures were used to discuss impacts of open wounds related to pelvic fractures. Here, a general but not limited to pelvic fractures, Gustilo-Anderson classification was used to determine open fractures [13]. In contrast, Faringer and Jones-Powell classifications [12, 14], which focused solely on location of the open wound and location of the open wound with fracture stability, respectively, were specifically carried out for open pelvic fractures.

Statistical analysis was conducted using SPSS version 18.0 (SPSS Inc., Chicago, IL, USA). Continuous and categorical variables are reported as mean and standard deviation and median and interquartile range, respectively. The cohort in this study was not normally distributed as found by Shapiro–Wilk test. Therefore, the study employed several nonparametric statistics analyses. Nonparametric Mann–Whitney U, Kruskal–Wallis, and Pearson’s chi-squared tests were performed on continuous dependent, ordinal, and categorical variables, respectively. Nonparametric Friedman test was used for comparison of three variables. Univariate and multivariate logistic regression was used to determine predictors of mortality. A two-tailed *P* value of 0.05 was considered statistically significant. In addition, post hoc power analysis was performed with the G*Power software (version 3.1; Franz Paul, University Kiel, Germany) to determine statistical power.

## Results

During the study period, 772 patients with pelvic fractures visited the emergency department (ED). Thirty-seven (4.9%) of whom were diagnosed with an open pelvic fracture. All patients with open pelvic fractures presented with shock and resuscitation procedures were immediately initiated upon their arrival. Eight patients expired during resuscitation at the ED due to multiple injuries. The overall mortality rate was 21.6%. After passing resuscitative procedures, 29 patients survived and were discharged after complete treatment courses.

Demographic data of the enrolled patients are shown in Table 1. The median age of the patients was 33 years (interquartile range (IQR) = 30.5), while the median ISS, NISS, and RTS were 27 (IQR = 17.5), 29 (IQR = 19), and 12 (IQR = 1.5), respectively. Among the injuries that accompanied the pelvic fractures, extremity fractures were the most common, followed by blunt chest injury and blunt abdominal injury. The survived patients were followed up at a mean of 15.7 months (IQR = 24).

**Table 1:** Demographic data of patients with open pelvic fractures

Total number of patients	37
Gender	
Male	23
Female	14
Age (years)	33 (IQR: 30.5)
Injury severity score (ISS)	27 (IQR: 17.5)
New injury severity score (NISS)	29 (IQR: 19)
Revised trauma score (RTS)	12 (IQR: 1.5)
Concomitant injuries	
Limb fractures	12
Blunt chest injury	10
Blunt abdominal injury	7
Urogenital injury	4
Spine injury	3
Head injury	2
AO/OTA fracture classification	
Type *A*	11
Type *B*	11
Type *C*	15
Gustilo Anderson classification	
Type I	5
Type II	9
Type III	23
Faringer classification	
Zone I	6
Zone II	18
Zone III	13
Jones-Powell classification	
Class I	11
Class II	5
Class III	21
Overall mortality rate	21.6%
Length of follow-up (survivors, *n* = 29, mons)	15.9 (IQR: 24)

IQR: interquartile range

**Fig. 1:**
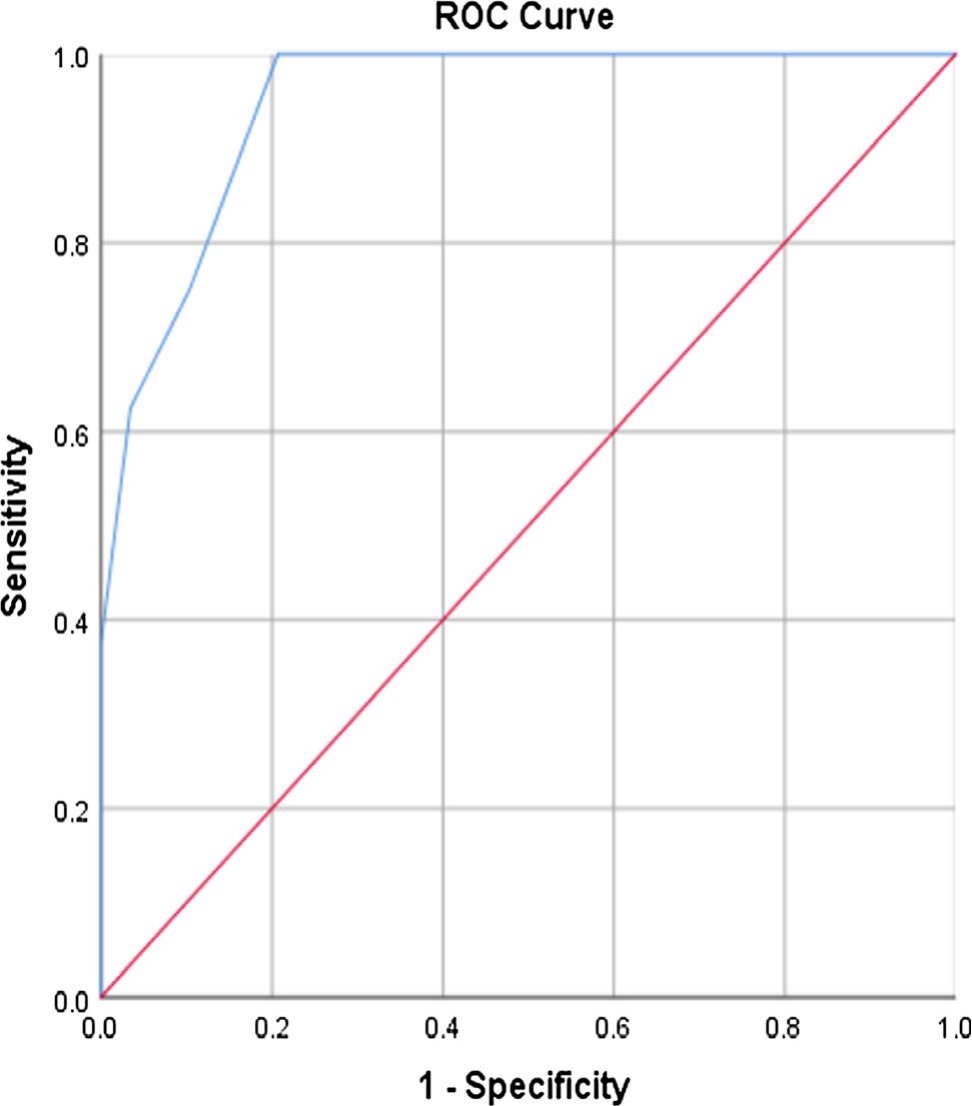
Receiver operating characteristic (ROC) curve for the predictive mortality in open pelvic fractures

To determine potential predictors that were directly related to a patient’s mortality after open pelvic fractures, univariate and multivariate logistic regression analyses were carried out. The chosen predictors and results of the univariate analysis are shown in Table 2. Using the full dataset, seven candidate predictors, namely ISS, NISS, RTS, Abbreviated Injury Scale (AIS)-head/neck, AIS-chest, AIS-abdomen, and AIS-extremity, revealed a significant result (*P* < 0.05) and were entered into the multivariate logistic regression analysis with mortality as the dependent predictor. Because a relative small number of patients were enrolled, a stepwise method of multivariate logistic regression analysis was applied, which resulted in only one significant independent predictor: RTS [*P* = 0.01, Odd’s ratio: 0.338 (0.148–0.773)]. The area under the curve (AUC) was 0.948 (95% confidence interval = 0.881–1.000, *P* < 0.01) on the receiver operating characteristic (ROC) curve (Fig. 1).

**Table 2:** Results of the univariate analysis for predictors of mortality

Predictors	Estimate	Standard error	*P* value	Odds ratio	95% confidence interval
Age	0.018	0.022	0.413	1.018	0.975–1.064
ISS	0.111	0.042	0.008^e^	1.117	1.030–1.212
NISS	0.127	0.050	0.012^e^	1.136	1.029–1.254
RTS	−1.083	0.421	0.01^e^	0.338	0.148-–0.773
Wound location^a^					
Class I (Reference)			0.68		
Class II	−0.095	1.338	0.943	0.909	0.066–12.524
Class III	0.654	1.215	0.591	1.923	0.178–20.819
Gustilo classification^b^					
Type I (Reference)			0.554		
Type II	−20.376	17,974.84	0.999	0.000	
Type III	−1.253	1.153	0.277	0.286	0.030–2.740
Jones-Powell classification			0.415		
Class I (Reference)	0.654	0.682	0.337	1.778	0.192–16.492
Class II	−0.732	0.563	0.193	0.444	0.073–2.7
Class III					
AIS-Head/Neck	0.540	0.244	0.027^e^	1.716	1.064–2.768
AIS-Face	−0.289	0.636	0.649	0.749	0.215–2.606
AIS-Chest	0.705	0.296	0.017^e^	2.023	1.131–3.617
AIS-Abdomen	0.723	0.301	0.016^e^	2.061	1.142–3.721
AIS-Extremity	1.590	0.578	0.006^e^	4.902	1.578–3.841
Major arterial injury^c^	−0.288	0.833	0.730	0.750	0.146–3.841
Fracture classification^d^	−1.897	1.174	0.106	0.150	0.015–1.497

^a^Farniger classification for wound location in open pelvic fracture [13]

^b^Gustilo-Anderson classification for open fractures [12, 14]

^c^From the results of angiography

^d^AO classification 2018

^e^statistically significant

We then used the results from the logistic regression analysis to obtain a scoring formula which predicted mortality after open pelvic fractures:

$$ {\text{Score}} = 10.012 + \left[ {{\text{RTS}}} \right]*\left( { - 1.083} \right) $$

$$ {\text{Youden index}}\, = \,0.089 $$

Using the generated score formula, we found that when the score was above −2, the mortality rate was 0%. However, when the score was below −2, the mortality increased to 100% (8/8).

## Discussion

In the current study, we analyzed a total of 37 patients during a 4-year period in a single medical center and generated a score formula in which RTS was identified as a single potential independent factor that affected acute mortality following open pelvic. Here, we found that the cutoff value of −2 was a predictable value to expect possible mortality after open pelvic fractures.

Open pelvic fracture is considered one of the most troublesome emergencies in trauma management [9]. Excessive retroperitoneal bleeding from the open wound could cause extremis; thus, hemostasis is a priority during resuscitation. Following aggressive blood transfusion, surgical debridement, external fixation, and osteosynthesis, bleeding from open wounds is controlled using surgical gauze packing. With our resuscitation protocol, mortality rate from open pelvic fractures was 21.6%, which was comparable to mortality rates found in recent literature [1, 8, 9, 11]. Apart from those who expired at the ED, no other patients died due to the injuries or subsequent complications such as sepsis. Those who survived resuscitation at the ED and underwent damage control orthopedic surgeries survived until the latest follow-up.

Several predictors have been reported to contribute to mortality in patients with open pelvic fractures as well as polytraumatized patients. One of such predictors could be the location of the open wound, although it is still debatable if it truly determines acute mortality in open pelvic fractures [9, 11, 14]. Faringer et al. first published their series on open pelvic fractures and postulated that the position of open wounds might affect mortality and therefore recommended elective diversional colostomy to reduce sepsis and mortality [14]. However, Giordano et al. recently reported that Faringer’s classification was not suitable for acute management of bony components in an open pelvic fracture [11]. In reported series, which is the current practice, early diversional colostomy has become a routine procedure in the early stages of open pelvic fractures, especially for Faringer zone I injury. On the other hand, Jones-Powell classification, developed in 1997, composed of the open wound site and fracture stability. Using this system, Cannada et al. [15] analyzed mortality of 64 patients with open pelvic fractures and found Jones-Powell class 3 in terms of perineal wound/rectal injury had the highest mortality rate. Nevertheless, the indication for diverting colostomy was unclear among surgeons. In addition, 4 patients with rectal injury (without diverting colostomies) died within 72 h due to trauma from hemorrhagic shock or cardiac arrest. In this study, we found that neither location nor size of the open wound is related to early mortality. This might be due to early diversional colostomy, which has been a routine procedure in early stages of open pelvic fractures, especially for Faringer zone I injury. We believe that aggressive debridement surgeries, early diversional colostomy for Faringer zone I and Jones-Powell class 3 injuries, and timely osteosynthesis could reduce risk of death in patients who survived resuscitation at the ED. In addition, emphasis on early diversional colostomy could prevent sepsis in these patient’s subacute or late, but not in early resuscitation stage.

Other predictors, such as age, gender, Gustilo classification for open fractures, AIS, major arterial injuries, and fracture classification, were also evaluated as potential predictors of death in open pelvic fractures. While male gender, severe multiple trauma, major hemorrhage, age, and presence of coagulopathy were previously reported as risk factors of mortality in pelvic fractures [16, 17], none of them has statistically contributed to the mortality in our group. Since we assumed patient population in our series had similar injury pattern: an open pelvic fracture, accompanied with severe and multiple organs' injuries, a single parameter could not be considered as potential predictor of mortality in such cases.

Therefore, a score system constituted a better predictor of mortality as it was composed of several parameters which could reflect various degrees of severity. Several score systems, such as the shock index, ISS, NISS, RTS, preexisting medical conditions, and trauma early prediction tool, were employed to expect mortality in polytraumatized patients [5, 17–20]. Classically, ISS, an anatomically based global severity scoring system that classifies injuries according to their relative gravity, was recognized as the indicator of mortality after blunt trauma [21]. Recently, researchers found that NISS, a modification of ISS, performs better in patients with multiple injuries than ISS [22, 23]. In addition, RTS, which is composed of Glasgow Coma Scale, systolic blood pressure, and respiratory rate, was also considered as a predictor of mortality in blunt trauma patients [9, 24–26]. Since these score systems were generally designed and applied on polytraumatized patients, we are interested in how these trauma scores affected death rate after open pelvic fractures. Here, multivariate logistic regression analysis found only RTS to potentially contribute to mortality after open pelvic fractures. Since all fatal cases presented deep shock status, we postulated this has weighted the impact of RTS during analysis. Based on the logistic regression analysis, we further developed a scoring model to predict the probability of mortality. One of the advantages of this equation is that it required only one parameter, RTS, which allowed for simpler calculations. Furthermore, the sum of the scores could be divided into two groups to distinguish the possibility of early mortality: below or above −2. To our knowledge, this is the first formula that is specifically used to evaluate the probability of early mortality in open pelvic fracture patients.

Since it was designed retrospectively, this study has many limitations. Firstly, incomplete medical records of patients during the analysis period could have affected the statistical results. Severe head injury and blunt abdominal trauma have been reported to be leading risk factors of mortality in polytraumatized patients [27–29], but our results did not reflect that, which might be due to the fact that our hospital is a referral trauma center. Patients with major traumas are likely to be resuscitated first at nearby satellite hospitals and subsequently handed over to our hospital. Therefore, those with extremis and had no chance of transferring might have had severe head and abdominal injuries. Secondly, blood loss from open pelvic fractures could not be correctly calculated, since bleeding in the trauma scene, during transit, and resuscitation at the ED could have been underestimated. However, a large volume of blood loss might result in decreased systolic blood pressure, which is one of RTS parameters. Therefore, we believe the score formula presented here is a credible method of predicting mortality within this cohort. Lastly, the sample size of this study was limited, which could give rise to bias during statistical analysis. In multivariate logistic regression analysis, the stepwise method could be applied due to the small sample size. Therefore, we did a post hoc analysis to detect the statistical power of this study. At the significance level of 0.05 and with a total sample size of 37, we obtained a 99% power for the scoring model to predict mortality. Nevertheless, further research is required to determine the clinical effectiveness of this formula.

In conclusion, mortality after open pelvic fracture remained a great concern. This study found that although the location of open wound might not be a causative etiology of early mortality, RTS could be a potential independent predictor of mortality. The scoring formula developed here could be used to assess mortality risk in open pelvic fracture patients during triage.

## References

[1] Hou Z, Smith WR, Strohecker KA (2012). Hemodynamically unstable pelvic fracture management by advanced trauma life support guidelines results in high mortality. Orthopedics.

[2] Keil DS, Gross S, Seymour RB (2018). Mortality after high-energy pelvic fractures in patients of age 65 years or older. J Orthop Trauma.

[3] Lunsjo K, Tadros A, Hauggaard A (2007). Associated injuries and not fracture instability predict mortality in pelvic fractures: a prospective study of 100 patients. J Trauma.

[4] Rittmeister M, Lindsey RW, Kohl HW (2001). Pelvic fracture among polytrauma decedents. Trauma-based mortality with pelvic fracture—a case series of 74 patients. Arch Orthop Trauma Surg.

[5] Gustilo RB, Mendoza RM, Williams DN (1984). Problems in the management of type III (severe) open fractures: a new classification of type III open fractures. J Trauma.

[6] Rothenberger D, Velasco R, Strate R (1978). Open pelvic fracture: a lethal injury. J Trauma.

[7] Bircher M, Hargrove R (2004). Is it possible to classify open fractures of the pelvis?. Eur J Trauma.

[8] Black EA, Lawson CM, Smith S (2011). Open pelvic fractures: the University of Tennessee Medical Center at Knoxville experience over ten years. Iowa Orthop J.

[9] Hermans E, Edwards MJR, Goslings JC (2018). Open pelvic fracture: the killing fracture?. J Orthop Surg Res.

[10] Fitzgerald CA, Moore TJ, Morse BC (2017). The role of diverting colostomy in traumatic blunt open pelvic fractures. Am Surg.

[11] Giordano V, Koch HA, Gasparini S (2016). Open pelvic fractures: review of 30 cases. Cases Open Orthop J.

[12] Jones AL, Powell JN, Kellam JF (1997). Open pelvic fractures. a multicenter retrospective analysis. Orthop Clin North Am.

[13] Gustilo RB, Anderson JT (1976). Prevention of infection in the treatment of one thousand and twenty-five open fractures of long bones: Retrospective and prospective analyses. J Bone Joint Surg Am.

[14] Faringer PD, Mullins RJ, Feliciano PD (1994). Selective fecal diversion in complex open pelvic fractures from blunt trauma. Arch Surg.

[15] Cannada LK, Taylor RM, Reddix R (2013). The Jones-Powell Classification of open pelvic fractures: a multicenter study evaluating mortality rates. J Trauma Acute Care Surg.

[16] Holstein JH, Culemann U, Pohlemann T (2012). Working group mortality in pelvic fracture patients. What are predictors of mortality in patients with pelvic fractures*?*. Clin Orthop Relat Res.

[17] Pandit V, Rhee P, Hashmi A (2014). Shock index predicts mortality in geriatric trauma patients: an analysis of the national Trauma data Bank. J Trauma Acute Care Surg.

[18] Coccolini F, Stahel PF, Montori G (2017). Pelvic trauma: WSES classification and guidelines. World J Emerg Surg.

[19] Kunitake RC, Kornblith LZ, Cohen MJ (2018). Trauma early mortality prediction tool (tempt) for assessing 28-day mortality. Trauma Surg Acute Care Open.

[20] Rogers FB, Osler T, Krasne M (2012). Has TRISS become an anachronism? a comparison of mortality between the national trauma data bank and major trauma outcome study databases. J Trauma Acute Care Surg.

[21] Dillon B, Wang W, Bouamra O (2006). A comparison study of the injury score models. Eur J Trauma.

[22] Staff T, Eken T, Wik L (2014). Physiologic, demographic and mechanistic factors predicting new injury severity score (NISS) in motor vehicle accident victims. Injury.

[23] Tohira H, Jacobs I, Mountain D (2012). Systematic review of predictive performance of injury severity scoring tools. Scand J Trauma Resusc Emerg Med.

[24] Alvarez BD, Razente DM, Lacerda DAM (2016). Analysis of the revised trauma score (RTS) in 200 victims of different trauma mechanisms. Rev Col Bras Cir.

[25] Jeong JH, Park YJ, Kim DH (2017). The new trauma score (NTS): a modification of the revised trauma score for better trauma mortality prediction. BMC Surg.

[26] Kondo Y, Abe T, Kohshi K (2011). Revised trauma scoring system to predict in-hospital mortality in the emergency department: glasgow coma scale, age, and systolic blood pressure score. Crit Care.

[27] El Mestoui Z, Jalalzadeh H, Giannakopoulos GF (2017). Incidence and etiology of mortality in polytrauma patients in a Dutch level I trauma center. Eur J Emerg Med.

[28] Radomski M, Zettervall S, Schroeder ME (2016). Critical care for the patient with multiple trauma. J Intensive Care Med.

[29] Shalhub S, Starnes BW, Brenner ML (2014). Blunt abdominal aortic injury: a Western Trauma association multicenter study. J Trauma Acute Care Surg.

